# Unilateral Pulmonary Artery Aplasia in a Pregnant Patient

**DOI:** 10.1155/2011/806723

**Published:** 2011-04-07

**Authors:** Chitra Lal, Jim Barker, Charlie Strange

**Affiliations:** ^1^Division of Pulmonary, Critical Care, Allergy, and Sleep Medicine, Medical University of South Carolina, 96 Jonathan Lucas Street, CSB 812, MSC 630, Charleston, SC 29425, USA; ^2^Scott & White Health System, Texas A&M HSC, Pulmonary 5C, 2401 S 31st Street, Temple, TX 76508, USA

## Abstract

Unilateral pulmonary artery aplasia is a rare anomaly. Case reports of this condition in pregnant patients are even more uncommon and the best approach to management of such patients is still unclear. We report a patient who presented with a history of dyspnea, chest pain, and hemoptysis. Imaging established the diagnosis in a newly pregnant female. Management of the pulmonary artery aplasia patient in pregnancy requires prospective evaluation of pulmonary hypertension.

## 1. Introduction

Unilateral pulmonary artery aplasia is a rare anomaly. Few case reports of this condition in pregnancy exist and the best approach to management of these patients is still unclear. We present a case report of a 31-year-old pregnant female who had an uneventful pregnancy. 

## 2. Case Report

A 31-year-old African-American woman presented to our clinic with a 5-year history of dyspnea and nonexertional retrosternal chest pain which radiated to the neck and upper back. Prior hemoptysis on two occasions 4 years ago was reported. She was a lifelong nonsmoker, used no illicit drugs, and denied any significant occupational exposures. 

**Figure 1:**
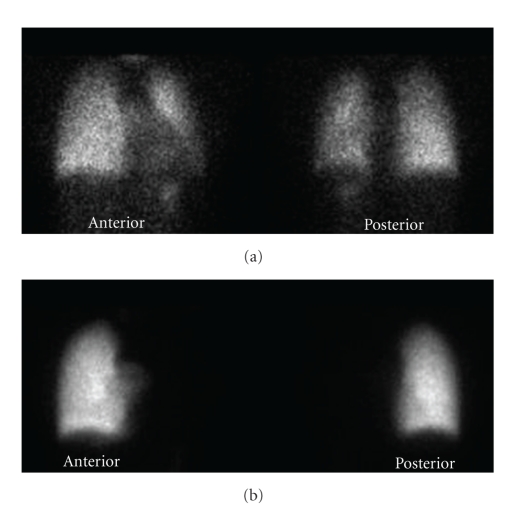
PA chest radiograph shows a small left lung volume with decreased vascular markings.

**Figure 2:**
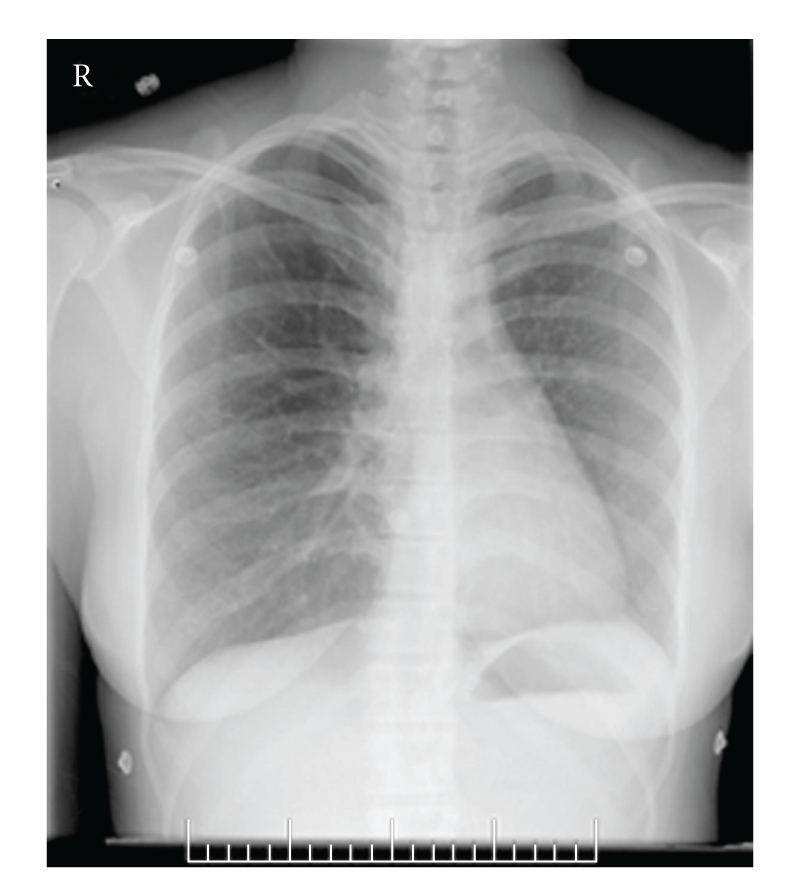
Ventilation-perfusion lung scan reveals bilateral ventilation and complete absence of perfusion in the left lung.

**Figure 3:**
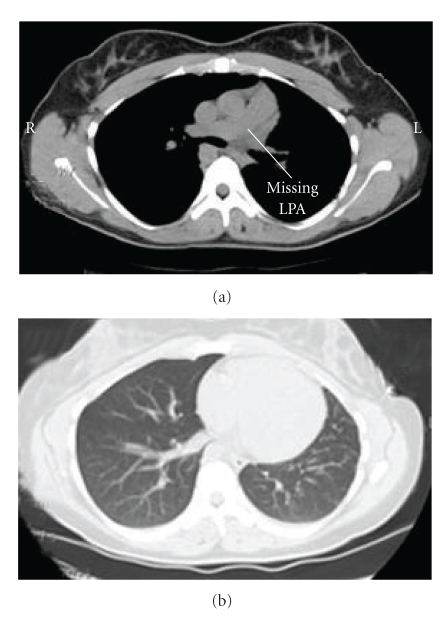
Noncontrasted chest CT shows a small left lung with left pulmonary artery aplasia. The right lung was normal.

Vital signs were normal with heart rate 84 bpm and blood pressure 100/68 mm Hg. Cardiac auscultation revealed normal heart sounds without a loud or split S2. A six minute walk test showed a walk distance walked of 900 feet with the lowest oxygen saturation recorded of 98%. Spirometry was normal. PA chest radiograph (Figure 1), ventilation-perfusion lung scan (Figure 2) as well as CT scan of the chest (Figure 3) established her diagnosis of left pulmonary artery aplasia. No other congenital abnormalities were seen.

A few days later, the patient reports that she is in the first trimester of pregnancy and is referred to high risk obstetrics-gynecology. 

An initial echocardiogram was normal revealing no tricuspid or pulmonic regurgitant jet, no right ventricular dilation, and an LVEF of greater than 55%. This is followed up with another echocardiogram 3 months later which again reveals no elevation of the pulmonary artery pressure. The pregnancy and delivery was uneventful. 

## 3. Discussion

Unilateral pulmonary artery aplasia is a rare anatomic anomaly that often is diagnosed in adulthood [1]. Retrospective case series have reported frequent pulmonary infections in 37%, dyspnea or exercise tolerance limitation in 40%, and/or hemoptysis in 20%. Pulmonary hypertension was seen in 44% of patients. 14 of 108 patients in this recent review were asymptomatic [1]. Chest pain and unilateral pleural effusion have also been described in a patient with unilateral pulmonary artery aplasia [2]. Although the initial diagnosis of pulmonary artery aplasia may be an incidental finding, during followup most patients develop symptoms. 

Lung perfusion is usually facilitated by formation of collateral vessels from the systemic circulation, most often the bronchial arteries. Since these arteries are perfused with systemic pressures and the alveolar structures of the lung are fragile, hemoptysis becomes an important symptom. If life threatening, embolization of collateral vessels may be considered. 

Pneumonectomy may be considered in patients with recurrent pulmonary infections of the affected side or in patients with recurrent or life-threatening hemoptysis. There have been case reports of surgical placement of a conduit to restore blood flow to the deficient lung in patients with pulmonary hypertension and congestive heart failure due to focal pulmonary artery agenesis [3]. 

One of the difficult issues for lifelong management of pulmonary artery aplasia patients is recognition and possible treatment of pulmonary hypertension (PH). Since the pulmonary vascular bed is reduced by only 50%, PH is not common in childhood in the absence of other congenital cardiac anomalies. Additionally, there may be compensatory vascular growth in the contralateral lung. However, unilateral lung perfusion with the entire cardiac output is a risk factor for pulmonary arterial hypertension. The mechanisms of this phenomenon are not completely clear, but may involve vascular sheer stress on the endothelium, injury from red blood cell products of hemolysis if present, and the tenuous state of having a vascular bed that is close to the 50% threshold for PH development. Since PH from pneumonectomy is rare, the exact threshold for PH development is unclear [4].

Case reports of pulmonary artery aplasia in pregnancy are extremely rare [5] and no clear guidelines regarding management of such patients exist. Pregnancy is known to increase cardiac output while impacting lung volume, both critical determinants for PH development. Therefore, the development of PH in pregnancy can be catastrophic and is associated with a high mortality. In women with known PH, it may be best to avoid pregnancy or terminate it at an early stage. If pregnancy is advanced, extensive evaluation and close monitoring is required. Although vasodilator therapy of PH in this condition has the potential to improve cardiac output, there are no case reports outlining drug use in secondary PH due to unilateral pulmonary artery aplasia in pregnancy. If considered, prudent use would mandate hospital observation during drug initiation. 

## 4. Conclusion

Pulmonary artery aplasia is a rare disease that can have a variable course during pregnancy. Diagnosis is established through imaging. Monitoring for PH is necessary to risk stratify female patients who desire childbirth. 

## References

[1] Derk Jan Ten Harkel A, Blom NA, Ottenkamp J (2002). Isolated unilateral absence of a pulmonary artery: a case report and review of the literature. *Chest*.

[2] Krall WR, Ploy-Song-Sang Y (1980). Unilateral pulmonary artery aplasia presenting with chest pain and pleural effusion. *Southern Medical Journal*.

[3] Toews WH, Pappas G (1983). Surgical management of absent right pulmonary artery with associated pulmonary hypertension. *Chest*.

[4] Deslauriers J, Ugalde P, Miro S (2011). Adjustments in cardiorespiratory function after pneumonectomy: results of the pneumonectomy project. *Journal of Thoracic and Cardiovascular Surgery*.

[5] Stiller RJ, Soberman S, Turetsky A, Lockwood C, Haddad R (1988). Agenesis of the pulmonary artery: an unusual cause of dyspnea in pregnancy. *American Journal of Obstetrics and Gynecology*.

